# 

**Published:** 1900-09-08

**Authors:** 


					THE HOSPITAL. v " The Standard Of highest purity."?Lancei, September Stii, 1900.
CADBURY'S ^ V ^ CADBURY'S
COGOA )?^ <MSk?> COCOA
wuwldtxlt rou, no drugs or alkalies used.
No. 72f Vol. XXVIII. ggggg Saturday,
September 8, 1900.
[SnbsCBTSp?s80?nt8'] PRICE twopence,
C!

				

